# Functional definition of endothelial progenitors by PROCR and PDGFRA co-expression

**DOI:** 10.1007/s10456-026-10078-0

**Published:** 2026-08-01

**Authors:** Cassandra Styke, Simranpreet Kaur, Seen-Ling Sim, Jilai Zhao, Chenhao Zhou, Ho Yi Wong, Jatin Patel, Edwige Roy, Mervyn C. Yoder, Richard P. Harvey, Abbas Shafiee, Kiarash Khosrotehrani

**Affiliations:** 1 The University of Queensland Frazer Institute, The University of Queensland, Brisbane, QLD 4102 Australia; 2 Mater Research—The University of Queensland, Brisbane, QLD 4101 Australia; 3 Queensland University of Technology, Brisbane, QLD 4101 Australia; 4 Department of Surgery, University of Pittsburgh, Pittsburgh, PA 15219 USA; 5 Victor Chang Cardiac Research Institute, Darlinghurst, NSW 2010 Australia; 6 School of Clinical Medicine, School of Biotechnology and Biomolecular Science, UNSW Sydney, Kensington, NSW 2052 Australia; 7 Metro North Hospital and Health Service, Brisbane, QLD 4029 Australia

**Keywords:** Refined endovascular progenitors, Endothelial cells, Vascularization, Lineage tracing, Regeneration

## Abstract

The functional and molecular definition of progenitors giving rise to blood vessel endothelium in vivo remains disputed. Upon investigating the overlap of seemingly divergent reports currently defining putative endothelial progenitor cells (EPCs) using single-cell RNA-sequencing and flow cytometry, Protein C Receptor (PROCR) and Platelet-Derived Growth Factor Receptor Alpha (PDGFRA) largely overlapped with previously characterized murine aorta’s CD34^+^CD31^low^ endovascular progenitors (EVPs). Functional assays and lineage tracing in homeostatic aorta and excisional wounds demonstrated increased clonogenic capacity, engraftment potential, and ability to form differentiated endothelial (D) cells of PROCR^+^ PDGFRA^+^ EPCs, termed as refined endothelial progenitor cell (rEPC), as compared to PROCR^neg^PDGFRA^neg^ EVPs. Similar *PROCR* and *PDGFRA* expression in normal human aorta, and increased clonogenic capacity of CD34^+^CD31^low^PROCR^+^ endothelial cells from freshly isolated human term placenta were observed as compared to controls. Functional validation of human rEPCs is supported by PROCR enrichment, while PDGFRA co‑expression in human endothelial progenitor–like cells is supported at the transcriptomic level only. Thus, overlapping PROCR and PDGFRA expression in EVPs narrows the population with true functional progenitor capacity.

**Supplementary Information:**

The online version contains supplementary material available at 10.1007/s10456-026-10078-0.

## Introduction

Vascularization occurs via *de novo* formation of new vessels (vasculogenesis or neovascularization) in an avascular tissue or the branching of existing vessels (angiogenesis), during embryonic development and in the adult [1–3]. It is an essential process for the growth of developing organs and the circulation of blood during tissue maintenance or repair [4]. Many studies have attempted to identify the progenitor cells that form the mature endothelial lining of blood vessels [1, 5]. It was debated whether these progenitors were of hematopoietic [1] or a mesenchymal lineage [6], whether they were bone-marrow derived, circulating or vessel-resident [7, 8] and, finally, whether they were unipotent or able to give rise to a variety of cell types in their progeny [9]. This has further complicated the functional criteria and cell surface markers to be used to define such a progenitor population [8, 10–15].

Over the past years, multiple populations with endothelial potential have been reported corresponding to the expected behavior of progenitors. A population of vascular endothelial stem cells positive for protein C receptor (PROCR) isolated from the mammary fat pad demonstrated higher colony formation, self-renewal and vessel-forming potential compared to PROCR^neg^ endothelial cells. In lineage tracing experiments, labelling of PROCR expressing cells resulted in endothelial clones forming tubes of endothelial cells but also NG2^+^ pericytes, likely deriving from the same clone suggesting a bipotential capacity [16]. In line with these findings and more recently, a rare circulating endothelial colony forming cell (C-ECFC) PROCR^high^CD34^bright^ population in the human cord blood, exhibited colony formation capacity and possessed in vivo vasculogenic function compared to the PROCR^neg^ counterpart, suggesting that C-ECFCs could potentially be enriched further based on PROCR expression [17]. Platelet-Derived Growth Factor Receptor Alpha (PDGFRA) is a potential marker for mesenchymal stem and progenitor cells, and recently has been associated with newly forming vasculature, appearing to increase in endothelial lineage commitment [18–21]. CD157 has been investigated as an additional endothelial stem cell marker, whereby CD157^hi^ endothelial side population (E-SP) isolated from the liver generated more endothelial cells in vitro and had clonal expansion and self-renewal capacity to generate functional blood vessels using serial and single-cell transplantation [22]. Importantly, this population was found to be a unipotent endothelial progenitor population, unable to generate cells of mesenchymal or hematopoietic lineage [22]. Another E-SP marked by ABCG2 also demonstrated similar progenitor activity with the potential to efflux Hoescht 33,342 [23]. Finally, transcription factor SOX18 has been thought to mark an endothelial progenitor population based on its transient expression in the endothelial cells of developing blood vessels [13].

Patel et al. termed this population the endovascular progenitor cells (EVPs, CD45^neg^CD34^+^VE-cadherin^+^CD31^lo^VEGFR2^lo^) [13]. This population showed colony formation capacity in limiting dilution in vitro as well as self-renewal capacity and engraftment potential in vivo as compared directly to other populations within the endothelial compartment. Moreover, lineage tracing experiments showed that EVPs had the ability to differentiate into mature differentiated endothelial (D) cells, characterized as being CD45^neg^CD34^+^VE-cadherin^+^CD31^+^VEGFR2^+^ over time. Based on these functional capabilities, EVPs were considered progenitors within the endothelial compartment. This work was translated into human models where a similar population was found in the umbilical cord blood and human term placenta that upon culture had all characteristics of endothelial colony forming cells (ECFCs) [9, 24]. This population was defined as CD45^neg^CD34^+^CD31^lo^ and formed high proliferative potential colonies (HPPs) in vitro. Limiting dilution colony formation and single-cell RNA sequencing analyses revealed that the EVP population is heterogenous, with many cells lacking self-renewal and colony formation ability. Therefore, true progenitors could be enriched further as only a fraction of the EVP population could self-renew [25].

We hypothesized that evaluating the overlap of the EVP population and previously reported endothelial progenitors markers, together with their respective functional capabilities outlined above from previous studies across the field will allow us to reach a more refined definition on murine EPCs and therefore improving our understanding of their biology and function. Here we first used single-cell RNA-sequencing and flow cytometry of the murine aorta to analyze the overlap of previously used putative EPC markers. Then we conducted immunofluorescence staining, functional assays, and lineage tracing experiments to confirm progenitor identity and functional capacity before finally translating these results to human models of aorta and term placenta. The results from this study provide a refined definition of EPC that addresses a longstanding knowledge gap in the fundamental biology of endothelial stem and progenitor cells and establishes a foundation for future mechanistic studies and therapeutic application.

## Materials and methods

### Single-cell RNA sequencing analysis

Single-cell RNA sequencing was analyzed using RStudio (RStudio, MA, USA) with the package Seurat (Version 4.2.0) [26]. Data were re-analyzed from previously conducted scRNA-seq of the murine aorta and publicly available scRNA-seq of the human aorta [25, 27]. We filtered out transcripts that were present in fewer than three cells. We removed outlier cells with fewer than 200 transcripts or more than 3000 transcripts. Cells expressing more than 20% mitochondrial genes were also excluded from downstream analysis. Data were then normalized using SCTransform method and integrated using the RPCA-based integration pipeline (2000 features as input to the anchor finding process) in Seurat to correct for batch effects. Principal component analysis was conducted using RunPCA on the integrated datasets. The first 30 principal components were used to compute nearest neighbors and clusters using FindNeighbors and FindClusters, respectively, with a resolution of 0.4 (mouse) or 0.5 (human) for optimal distinction between clusters.

Clustering plots were made using the two-dimensional Uniform Manifold Approximation and Projection (UMAP) algorithm in Seurat. Data was then log-normalized for identifying DE genes using “FindAllMarkers” function with default parameters. DE genes that were at least 0.25-fold difference (log-scale) between the two groups of cells were kept. Clusters were annotated using the Bioconductor package SingleR with reference to the Mouse RNA Sequencing Data and Human Primary Cell Atlas Data databases, respectively [28–30].

### Animals

All mice were treated in accordance with University of Queensland ethics approvals and conformed to the regulations set out in the Australian Code for the Care and Use of Animals for Scientific Purposes (8th Edition) and the Animal Care and Protection Act 2001. Mixed sex adult C57Bl/6 mice and NOD *scid Il2ry*^*null*^*B2m*^*null*^ (NSG) mice were obtained from the Animal Resources Centre (Western Australia, Australia). Mixed sex adult *CAG-EGFP*, *Cdh5-Cre*^*ERT2*^*/ROSA-EYFP*,* Pdgfrα-MerCreMer/ROSA-YFP*,* Abcg2-Ires-CreERT2/ROSA-YFP* and *Sox18-Cre/ROSA-YFP* mice were supplied from in-house breeding colonies.

*Cdh5-Cre*^*ERT2*^*/ROSA-EYFP*,* Cdh5-Cre*^*ERT2*^*/ROSA-ZsGreen*,* Pdgfrα-MerCreMer/ROSA-YFP*,* Abcg2-Ires-CreERT2/ROSA-YFP* and *Sox18-Cre/ROSA-YFP* mice were injected (intraperitoneal) with 100 µL of 20 mg/mL Tamoxifen (Sigma-Aldrich, MI, USA) in 90% corn oil (Sigma-Aldrich) and 10% ethanol for 5 consecutive days to induce recombination of the yellow fluorescent protein (YFP) in target cells prior to tissue collection (only 3 days of injections were used for *Cdh5-Cre*^*ERT2*^*/ROSA-ZsGreen* mice as per standard protocol). Mice were euthanized by carbon dioxide asphyxiation in accordance with the University of Queensland’s ethical guidelines.

### En Face/Aorta length preparation

Aortae were dissected and prepared for *en face* IF as previously described by Zhao et al. [31]. The resulting dissected aorta was coated in optimal cutting temperature (OCT) solution (Sakura Finetek, CA, USA), and rolled downward from thoracic to abdominal aorta before being embedded in OCT and snap-frozen in 100% ethanol on dry ice to be stored at − 30 °C. Samples were sectioned perpendicularly to the cut face in order to analyze the length of the aorta.

### Immunofluorescence

Tissues were prepared and stained as described previously [31]. Briefly, samples were optionally permeabilized (depending on antibody) in 0.1% Triton X-100 (Sigma-Aldrich, MO, USA) in 1x PBS and incubated in a blocking solution containing 3% bovine serum albumin (BSA; Sigma-Aldrich, MO, USA) and 20% normal goat serum (NGS; Vector Laboratories, CA, USA) in PBST for 45–60 min at room temperature before staining in a solution containing primary antibodies in 3% BSA/PBST overnight at 4 °C or at room temperature for 1 h. In this study, primary antibodies used included rat anti-mouse CD31 (1:100; BD Biosciences), rabbit anti-mouse CD34 (1:100; Abcam), rabbit anti-mouse ERG (1:100; Abcam), rat anti-mouse PROCR (1:200; Invitrogen), chicken anti-GFP (1:200; Invitrogen), and Griffonia (Bandeiraea) Simplicifolia Lectin I (GSL I, BSL I)—Rhodamine (Isolectin, 1:50; Vector Laboratories). Secondary antibodies conjugated with Alexa-fluor 488, 568, or 647 (Invitrogen, Carlsbad, CA, USA) were used for fluorescence detection., and DAPI (Invitrogen, CA, USA) was used for staining of nuclei. Fluorescence imaging was performed using an Olympus FV3000 confocal microscope (Olympus, Shinjuku, Japan) and a Nikon/Spectral Spinning Disc confocal microscope (Nikon, New York, USA). Brightfield imaging was conducted using a Nikon Eclipse 50i Brightfield Microscope (Nikon, New York, USA). Image analysis was conducted using the Olympus Fluoview FV31S-SW software (Olympus, Shinjuku, Japan) as well as ImageJ (National Institute of Health).

### Flow cytometry and fluorescence-activated cell sorting

Aortae and full-skin excisional wounds were digested as described previously [31]. For comparison of colony formation between thoracic and abdominal aorta, aortae were divided at the diaphragm. Antibodies used to assess the endothelial hierarchy and subpopulations included, in murine aorta and full-skin excisional wounds respectively: Hematopoietic Lineage Cocktail PerCP-Cy5.5 (1:50, 1:300), VE-Cadherin BV421 (1:100, 1:25), CD34 Alexa Fluor 647 (1:150, 1:100), CD31 PE-Cy7 (1:1000, 1:300), PROCR PE (1:200, N/A), PDGFRA BV605 (1:100, 1:50), CD157 PE (1:400, N/A), and 7-AAD PE-Cy5 (Live/Dead; 1:100, 1:100). VEGFR2 expression closely mirrored CD31 expression across endothelial populations [13] and does not provide additional discriminatory power for isolating the progenitor population of interest. Therefore, VEGFR2 was omitted from the final gating strategy to simplify the panel while retaining equivalent endothelial specificity. Antibodies were obtained from BD Bioscience or BioLegend; PROCR antibody was obtained from Invitrogen.

Flow cytometry analysis was conducted on an LSR Fortessa flow cytometer (BD Biosciences, CA, USA) and fluorescence-activated cell sorting was conducted on a FACSAria Fusion Sorter (BD Biosciences, CA, USA). Single stain controls were used to acquire cytometer voltage settings and to compensate data. Fluorescence-minus-one (FMO) controls were used to distinguish positive and negative populations and set appropriate gates. All analysis was conducted using FlowJo^®^ software (FlowJo LLC, USA).

### In vitro colony formation assay

Matrigel^®^ (Corning^®^ Matrigel^®^ Basement Membrane Matrix, Phenol Red-free, LDEV-free; Corning, New York, USA) was deposited into each well of 96-well plates and incubated at 37 °C for 1 h for gelation to occur. This method allows Matrigel to be used as a thin coating substrate to facilitate cell attachment, and not as a tube‑formation or capillary‑like network assay. Cells from fluorescence-activated cell sorting and Endothelial Growth Medium-2 (EGM2; Lonza, Basel, Switzerland) were then deposited on top of cross-linked Matrigel^®^. Cells were divided to allow the deposition of 10 or 100 cells into each well, depending on experimental conditions. Plates were then incubated at 37 °C and media was replaced twice weekly. Cells were imaged intermittently using a Nikon Eclipse 50i Brightfield Microscope (Nikon, New York, USA).

On day 12, wells were fixed in 4% PFA, then permeabilized in 1X PBSTx before blocking in 3% BSA/10% NGS in PBST. Cells were then stained with primary antibody solutions in concentrations outlined above overnight at 4 °C. The following day, cells were incubated in secondary antibody solutions and stained with a 1:5000 solution of DAPI before imaging as described above.

### In vivo vessel generation and collagen plugs

Following FACS sorting, 100 cells of desired populations isolated from *CAG-EGFP* mice were mixed with gel solutions prepared on ice by mixing 80% collagen (3% PurCol; Advanced Biomatrix, Carlsbad, CA, USA) with 10% DMEM, 5–7% sodium bicarbonate to reach a pH of 7.2–7.4, and water. Gel + cell solutions were incubated in 96-well plates at 37 °C for 90–120 min before topping with EGM2 and incubating overnight.

The following day, gels were rinsed in PBS and implanted into NOD-*scid Il2ry*^*null*^*B2m*^*null*^ (*NSG*) mice. Lateral incisions were made on each dorsal flank to create a pocket below the skin and above the muscle, with a total of two plugs being implanted into each mouse. Incisions were sutured and mice were monitored daily. Plugs were collected after 7 days and analyzed using wholemount microscopy as well as cryosections for IF. Quantification of collagen plug assay sections was performed using ImageJ software (NIH). Images were analyzed in a blinded manner and were quantified and averaged for downstream analysis. For wholemount microscopy, gels were rinsed in PBS before clearing in RapiClear^®^ (SunJin Lab Co, Taiwan) for 30–60 min and imaging immediate. Gels were then subject to a sucrose gradient as described above to prepare for cryo-sectioning.

### Human term placental cells

Frozen single cells suspensions of human term placenta samples previously processed as per the protocol outlined in Nano et al. were thawed and prepared for flow cytometry/FACS-sorting as previously described [32]. In addition to the markers outlined in this previously published protocol, PROCR PE (1:25; BioLegend) was added to the panel. Cells were then gated and sorted as previously described, with the addition of a PROCR^+/neg^ gate on each of the 4 populations of varying CD31 expression.

Cells that were FACS-sorted were then plated onto collagen-coated plates as previously described at a 1000 cells per well density in 48-well plates with EGM2. Cells were cultured and allowed to expand for up to 30 days to evaluate colony formation capacity (no colony, endothelial colony (EC, < 50 cells), low-proliferative potential ECFC (LPP-ECFC, < 1000 cells) or high-proliferative potential ECFC (HPP-ECFC, > 1000 cells, ability to form secondary colonies)). HPPs were then further passaged to limiting dilution assay conditions (1000 cells per well in a 6-well plate) for evaluation of further colony formation, serially passaged for expansion, or stained to evaluate immunofluorescent expression using rabbit-anti-human VECAD (1:100; BD Biosciences) and mouse-anti-human CD31 (1:100; BD Biosciences). The placental work was approved by the institutional human research ethics committee (ethics number: HREC/09/QRBW/14). The cells pooled from 8 donors (Third trimester, Caucasians) and 3 independent repeats of experiments were conducted.

### Statistical analysis

Data were analyzed using GraphPad Prism8 (GraphPad, United States) software. Paired t-tests and unpaired t-tests for single comparison results, depending on experimental conditions. For multiple comparisons, Friedman one-way ANOVA, two way ANOVA, and Kruskal-Wallis tests were conducted in accordance with data. Results were shown as an average with error bars representing the standard deviation (SD), with a significance threshold set at *p* < 0.05. A minimum of three biological replicates were used for all significance testing.

## Results

### Single-cell RNA-sequencing reveals key markers in endothelial populations

**Fig. 1:**
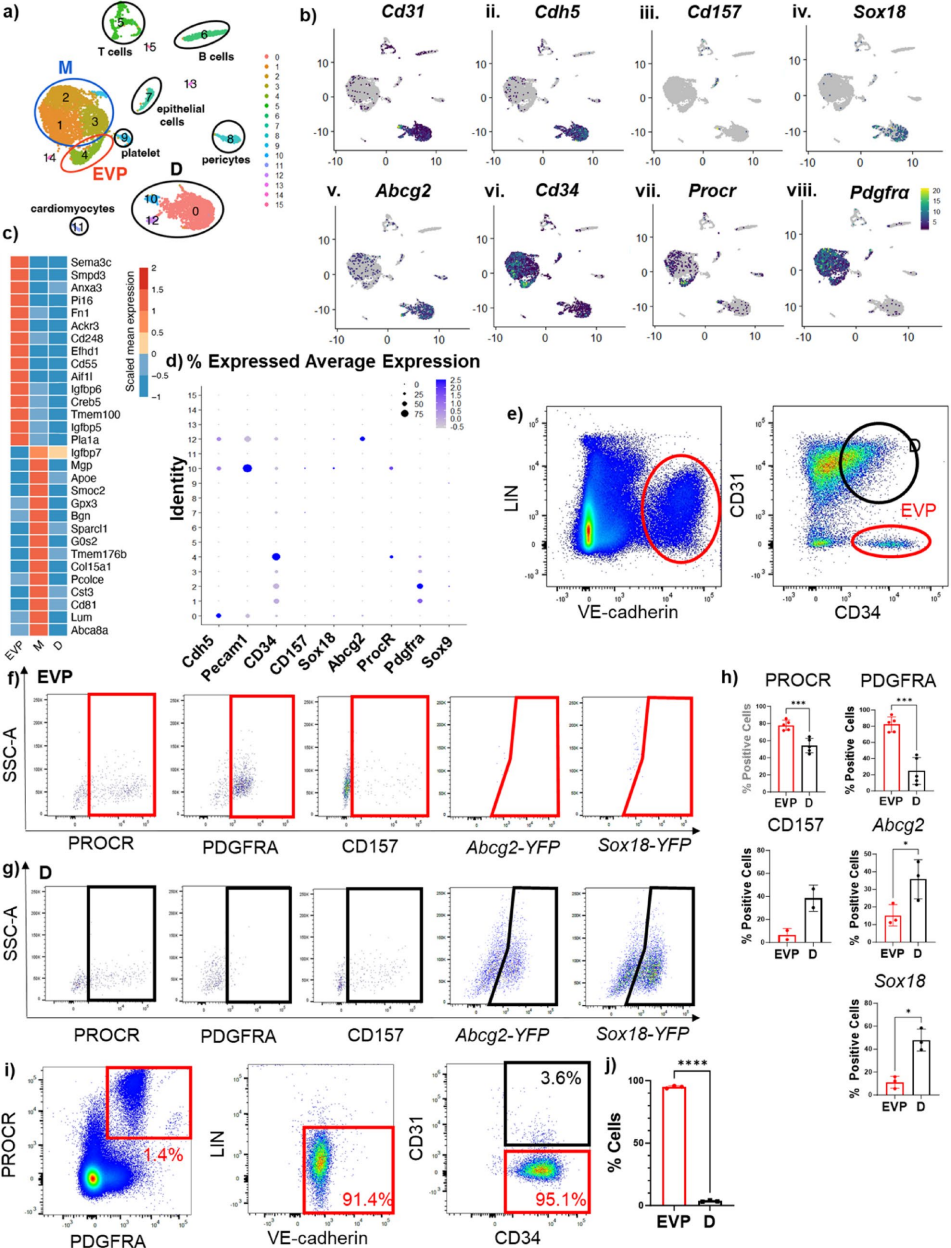
Single-cell RNA-sequencing and flow cytometry reveal endothelial protein C receptor (PROCR) and platelet derived growth factor – A (PDGFRA) as key markers in endothelial populations. **a** UMAP plot shows different subclusters of endothelial (Lin^neg^CD34^+^) cells from murine aorta (*n* = 3). Endovascular progenitors (EVP), differentiated (D) and mesenchymal (M) cell clusters were highlighted for our clusters of interest. **b** Expression levels of genes of interest overlaid on the UMAP plot highlighting the cluster specificity of their expression. The expression levels are shown as log2(counts + 1)-transformed values, and the gradient represents low (purple) to high (yellow) values. **c** Heat map showing top 30 markers (15 UP; 15 DOWN) for EVP (Cluster 4) vs. M (Cluster 1/2/3) vs. D. **d** Dot plot showing relative expression of genes of interest across clusters. The size of dots represents the percentage of cells in each cluster that have non-zero expression of each gene. The color gradient indicates mean expression level of all cells in each cluster. **e** Representative flow cytometry dot plots showing the gating strategy used to isolate the endothelial hierarchy. Endothelial cells were gated as Lineage (Lin)^neg^VE-cadherin^+^. From this population, EVP cells were gated as the CD31^neg/lo^CD34^+^ population whereas D cells were gated as the CD31^+^CD34^+^ population. **f**, **g** Representative flow cytometry dot plots showing expression of Procr, CD157, Abcg2-YFP, and Sox18-YFP in (**f**) EVP and (**g**) D cells. **h** Quantification of expression of cell surface markers (**i**) Procr (*** *p* = 0.0008; *n* = 5), (ii) PDGFRα (*** *p* = 0.0005; *n* = 5), (iii) CD157 (*n* = 2 descriptive observation), (iv) Abcg2 (*p = 0.0490; *n* = 3), and (v) Sox18 (*,*p* = 0.0452; *n* = 3) in EVP and D cells **i** Representative flow cytometry dot plots showing alternative gating strategy where live cells are first gated as PROCR^+^PDGFRA^+^, followed by Lin^neg^VE-cadherin^+^ and finally gated as EVP and D cells based on CD31 and CD34 expression. **j** Quantification of percent of EVPs gated using gating strategy in (**i**) (****, *p* < 0.0001, *n* = 3). Data are presented as mean ± SD. Statistical analysis was performed using paired t-test

Previously conducted single-cell RNA-sequencing on the Lin^neg^CD34^+^ compartment of the aortae of three *C57Bl/6* mice was re-analyzed to illuminate highly expressed genes in endothelial and mesenchymal clusters of interest (Supplementary Fig. 1a) [25]. To better capture the transcriptomic heterogeneity among endothelial subpopulations, we applied a higher resolution when performing unsupervised clustering. More distinctive Lin^neg^CD34^+^ cell subclusters emerged, allowing the analysis of gene expression within previously delegated clusters (Fig. 1a).

Clusters 0, 10, and 12 were characterized as mature differentiated endothelial (D) cells based on expression of pan-endothelial markers classically used to define the endothelial compartment including *Pecam1* (CD31) and *Cdh5* (CD144), confirmed using SingleR labeling analysis (Fig. 1a and b (i-ii), Supplementary Fig. 1b). Clusters 1, 2, and 3 showed upregulation of mesenchymal markers leading to their designation as mesenchymal (M) clusters, whereas cluster 4 showed expression of both mesenchymal and endothelial markers, leading to the designation of this cluster as the putative endovascular progenitor (EVP) population (Fig. 1a). Neither the putative mesenchymal or EVP groups possessed expression of hematopoietic cell markers (confirmed with SingleR, data not shown).

Cluster 4 maintained key endothelial marker expressions such as Cdh5, Pecam1 or CD34 although at a lower level compared to differentiated endothelial cells (Supplementary Fig. 1). Among candidate progenitor genes studied, *Cd157* (Cluster 10), *Sox18* (Cluster 0, Cluster 10), and *Abcg2* (Cluster 0, Cluster 12) showed significant upregulation (adj *p* < 0.001) in the differentiated endothelial cell clusters (Fig. 1b (iii-v)) while *Procr* (Cluster 4) and *Pdgfrα* showed expression in EVP cluster 4, with mesenchymal marker *Pdgfrα* being most upregulated in the mesenchymal M cell clusters (Cluster 1, Cluster 2; Fig. 1b (vi-viii)). Heat map analysis of top 30 markers in EVP cluster 4 showed upregulation of genes expressed in endothelial cells (Fig. 1c). Pathway analysis and dot plots of genes of interest conducted on cluster 4 to characterize differentially expressed (DE) genes showed enrichment of angiogenic, TGFB- and Wnt-signaling pathways, and genes related to both vascular maintenance and development (*Sema3c*,* Tmem100*,* Mfap5*) and mesenchymal populations (*Igfbp5*,* Igfbp6*,* Pcolce2*), potentially indicating that the population maintains an identity balanced between endothelial and mesenchymal states (Fig. 1d, and Supplementary Fig. 1c-f). Other major clusters comprised non-endothelial cell populations, including some remaining hematopoietic cells not depleted during the flow sort particularly T cells (C5; Cd3g, Cd3d, Cd3e), B cells (C6; Cd19, Cd79a) but also epithelial cells (C7; Krt19, Krt18, Krt7), pericytes (C8; Rgs5, Pdgfrb), platelets (C9; Pf4, Gp1bb), and cardiomyocytes (C11; Myl7, Myoz2) (Fig. 1a).

### EVPs highly express PROCR and PDGFRA

To narrow a true EPC population, flow cytometry was performed on adult *C57BL/6* mouse aortae using the markers identified in above single-cell RNA sequencing analysis in conjunction with the previously used markers to characterize EVPs [25]. From total aorta cells, the endothelial hierarchy was segregated based on cell surface marker profiles and the original gating strategy outlined in Patel et al. [13]. Putative EVPs were segregated as Lin^neg^VE-cadherin^+^CD34^+^CD31^neg/lo^, and D cells as Lin^neg^VE-cadherin^+^CD34^+^CD31^+^ (Fig. 1e). EVP and D cells were then further evaluated for expression levels of putative endothelial progenitor markers PROCR [16], PDGFRA [20], and CD157 [22] (Fig. 1f-g, Supplementary Fig. 2a). *Procr* and *Pdgfra* were 1.4-fold (*p* < 0.001) and 3.3-fold (*p* < 0.001) more frequently expressed in EVPs as compared to D cells, respectively, whereas *CD157* was 5.9-fold (descriptive observation.) more highly expressed in D cells than in EVPs (Fig. 1f-h, Supplementary Fig. 2b). In addition to these markers, two additional mouse strains were used based on the studies described above to test for differences in *Abcg2* [33] and *Sox18* [13] between EVP and D cells.

Flow Cytometry analysis was performed on aortae of adult *Abcg2-Ires-Cre*^*ERT2*^*/ROSA-EYFP* [33] and *Sox18-Cre* ^*ERT2*^*/ROSA-EYFP* [13] mice treated with tamoxifen for 5 consecutive days. Characterization of EVP and D populations based on YFP expression showed that *Abcg2* and *Sox18* were 2.3-fold (*p* < 0.05) and 4.3-fold (*p* < 0.05) more frequently expressed in D cells compared to EVPs, respectively (Fig. 1f-h).

These findings suggested that PDGFRA and PROCR were additional markers that could enrich progenitors within the already described EVP population. Interestingly, among EVP cells, an average of 78.04% were PROCR^+^ and 82.28% were PDGFRA^+^, suggesting that these markers may allow refining of the progenitor definition. Importantly, an alternative gating strategy on live aortic cells co-expressing both PROCR and PDGFRA showed that an average of 91.43% (** *p* = 0.0012) were Lin^neg^VE-cadherin^+^, and from here a further 94.97% (**** *p* < 0.0001) were CD34^+^CD31^neg/lo^, corresponding to EVPs (Fig. 1i; *n* = 3). This demonstrates the powerful ability of PROCR and PDGFRA co-expression alone to label the same population as the classic EVP gating strategy to a high degree of confidence. Similarly, the large overlap between PROCR and PDGFRA among EVPs allowed us to use only one marker at a time to examine functional characteristics.

Given the significant overlap of three distinct strategies to identify progenitor cells in the endothelium, we henceforth called this population a refined endothelial progenitor cell (rEPC) population and proceeded to its functional analysis. To avoid ambiguity, we explicitly define rEPCs as non‑hematopoietic, endothelial‑restricted progenitors, distinct from classical EPCs described in Asahara et al. The use of single-cell RNA-seq suggested that cluster 4 was representative of this cell population. Differential gene expression defining this cluster included the expression of both major endothelial and mesenchymal genes as seen in the analyses of top differentially expressed genes from this cluster including *Procr* and *Pdgfra* (Supplementary Fig. 1c, 1e).

### rEPCs show increased endothelial colony formation capacity in vitro and increased engraftment potential in vivo

**Fig. 2:**
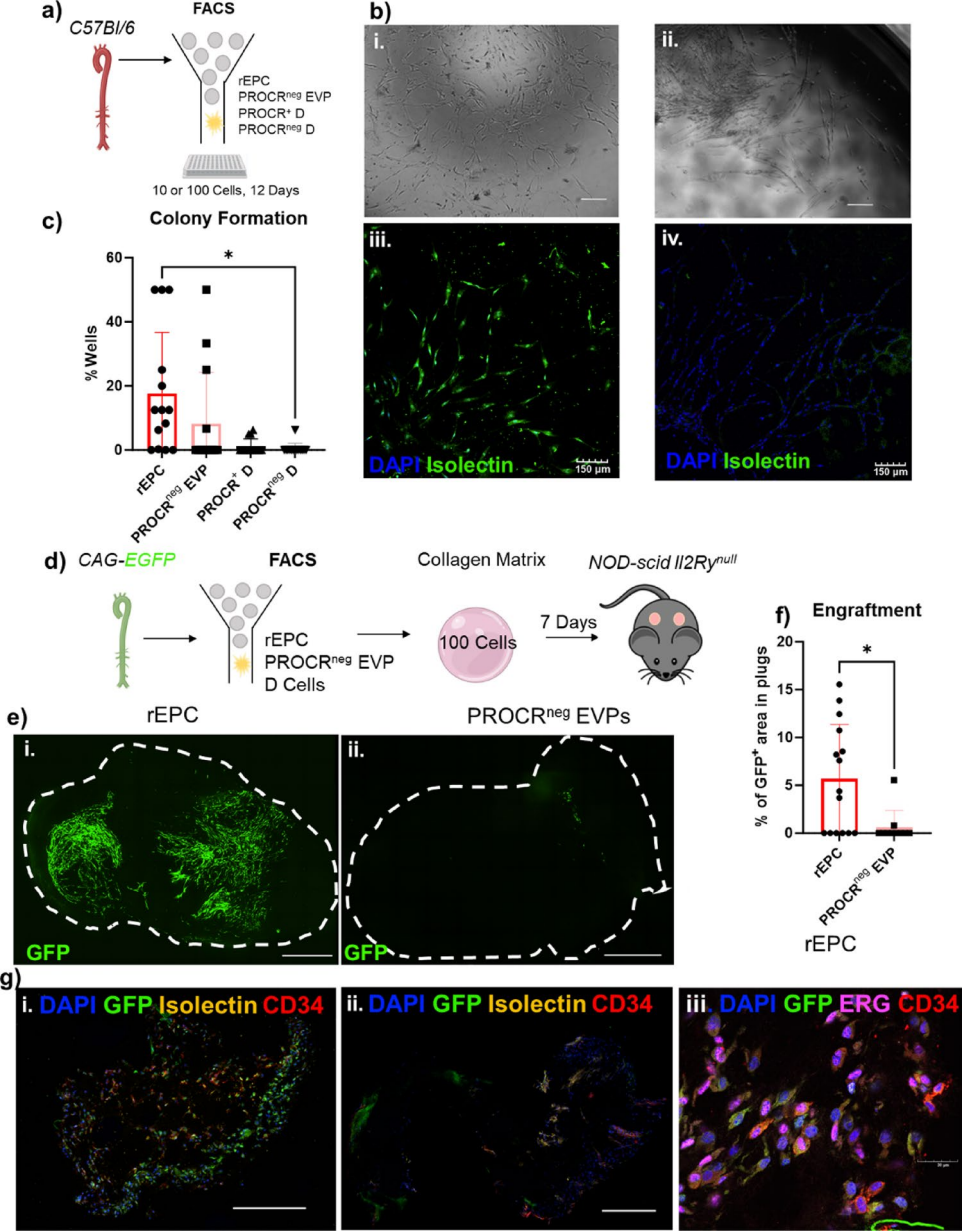
Refined endothelial progenitor cells (rEPCs) show increased endothelial colony formation capacity in vitro and engraftment potential in vivo compared to other populations. **a** Endothelial cells from C57Bl/6 aorta were FACS sorted based on cell surface expression of PROCR as depicted in graphical representation. Briefly, the live lineage (Lin)^neg^V-CADH^+^CD34^+^ cells gate first from which CD31^neg/lo^PROCR^+^ rEPC, CD31^neg/lo^PROCR^neg^ endothelial vascular progenitors (EVPs), CD31^+^PROCR^+^ differentiated (D) cells, and CD31^+^PROCR^neg^ D cells were sorted. **b** Representative brightfield images (**i-ii**) depicting endothelial (**i**) and elongated (**ii**) cell morphology types at day 12 (4x magnification; scale bar = 500 μm) and immunofluorescence (IF) staining (**iii-iv**) of endothelial (**iii**) and elongated mesenchymal like (**iv**) colonies on day 12 showing expression of Isolectin BSL-I (10x magnification; scale bar = 150 μm). **c** Percentage of colonies formed in each condition based on number of wells plated with all experiments normalized to 10 cells per well (*n* = 14; * *p* < 0.05). **d** Graphical representation depicting experimental procedure for in vivo collagen gel engraftment assay. **e** Representative images of collagen gels containing (**i**) rEPCs or (**ii**) PROCR^neg^ EVPs FACS-sorted from CAG-EGFP mice aortae collected following 7 days of implantation in NOD-scid Il2ry^null^B2m^null^ (NSG) mice (scale bars = 500 μm). **f** Percentage of GFP^+^ area of each gel upon collection measured via IF (* *p* < 0.05). **g** Representative IF images of sections from (i) rEPC and (ii) PROCR^neg^ EVP collagen gels collected after 7 days and stained with DAPI, GFP, CD34 and Isolectin showing colocalization of GFP, Isolectin and CD34 (scale bars = 250 μm) and (iii) rEPC showing colocalization of GFP, ERG and CD34 (60x). a and d created with BioRender.com. Data are presented as mean ± SD. Statistical analysis was performed using Friedman one-way ANOVA (c) and unpaired t-test (**f**)

To analyze the functional capacity of rEPCs versus other EVP and D cell populations, PROCR+ EVPs (rEPCs), PROCRneg EVPs, PROCR + D cells, and PROCRneg D cells were sorted from adult C57Bl/6 aorta and cultured in Matrigel for colony forming assay (Fig. 2a). The percentage of colonies formed in each condition based on number of wells plated with all experiments normalized to 10 cells per well was calculated (*n* = 14; * *p* < 0.05). rEPCs possessed the greatest colony formation capacity with a mean of 18% of wells plated per mouse displaying colonies, followed by 8% of PROCR^neg^ EVP wells (Fig. 2c i-ii; *, *p* < 0.05). Among the colonies formed, two major morphologies were seen at day 12: a classic endothelial morphology (Fig. 2b (i)) with positive expression of endothelial marker as revealed by Griffonia (Bandeiraea) Simplicifolia Lectin I (GSL I, BSL I) (Isolectin) (IF; Fig. 2b (iii)), and an elongated morphology (Fig. 2b (ii)) that was not labeled by Isolectin (Fig. 2b (iv)).

rEPCs formed exclusively isolectin + colonies with endothelial morphology, while PROCR^neg^ EVPs formed isolectin negative colonies with an elongated morphology. The ability of rEPCs to give rise to endothelial cells reflect rEPCs as true progenitor cells. Importantly, D cells (PROCR^+^ or PROCR^neg^) never formed endothelial colonies based on positive staining for Isolectin.

To distinguish the potency of rEPCs and PROCR^neg^ EVPs, these populations were challenged with a more stringent in vivo assay where collagen gels containing no cells (control), 100 rEPCs, PROCR^neg^ EVPs, or total D cells, respectively, from *CAG-EGFP* mice were transplanted into the dorsal flanks of *NOD-scid-Il2ry*^*null*^*B2m*^*null*^ (*NSG*) recipients (Fig. 2d). Whole mount images of collagen gels after 7 days revealed that rEPCs had the highest engraftment potential with 9/15 gels engrafting and an average of 5.7% GFP^+^ area per plug (Fig. 2e (i), 2f), while only 2/10 PROCR^neg^ EVP gels engrafted with an average of 0.8% GFP^+^ area per plug (Fig. 2e (ii), 2 F; *p* < 0.05). D cells were never able to engraft (0/6 gels), showing 0% GFP^+^ area, identical to the results of the gels containing no cells. IF staining was conducted on sections of these gels to further characterize the cells that had engrafted. GFP^+^ rEPCs co-expressed endothelial markers CD34, ERG and Isolectin while no overlap of these endothelial markers was seen with GFP^+^ cells from collagen gels containing PROCR^neg^ EVPs (Fig. 2g). These findings more robustly pointed to key functional differences between EVP and D cells as reported [34, 35], but more remarkably between rEPCs expressing PROCR and EVPs devoid of PROCR.

### rEPCs form a niche in the thoracic aorta and display increased clonogenic capacity

**Fig. 3:**
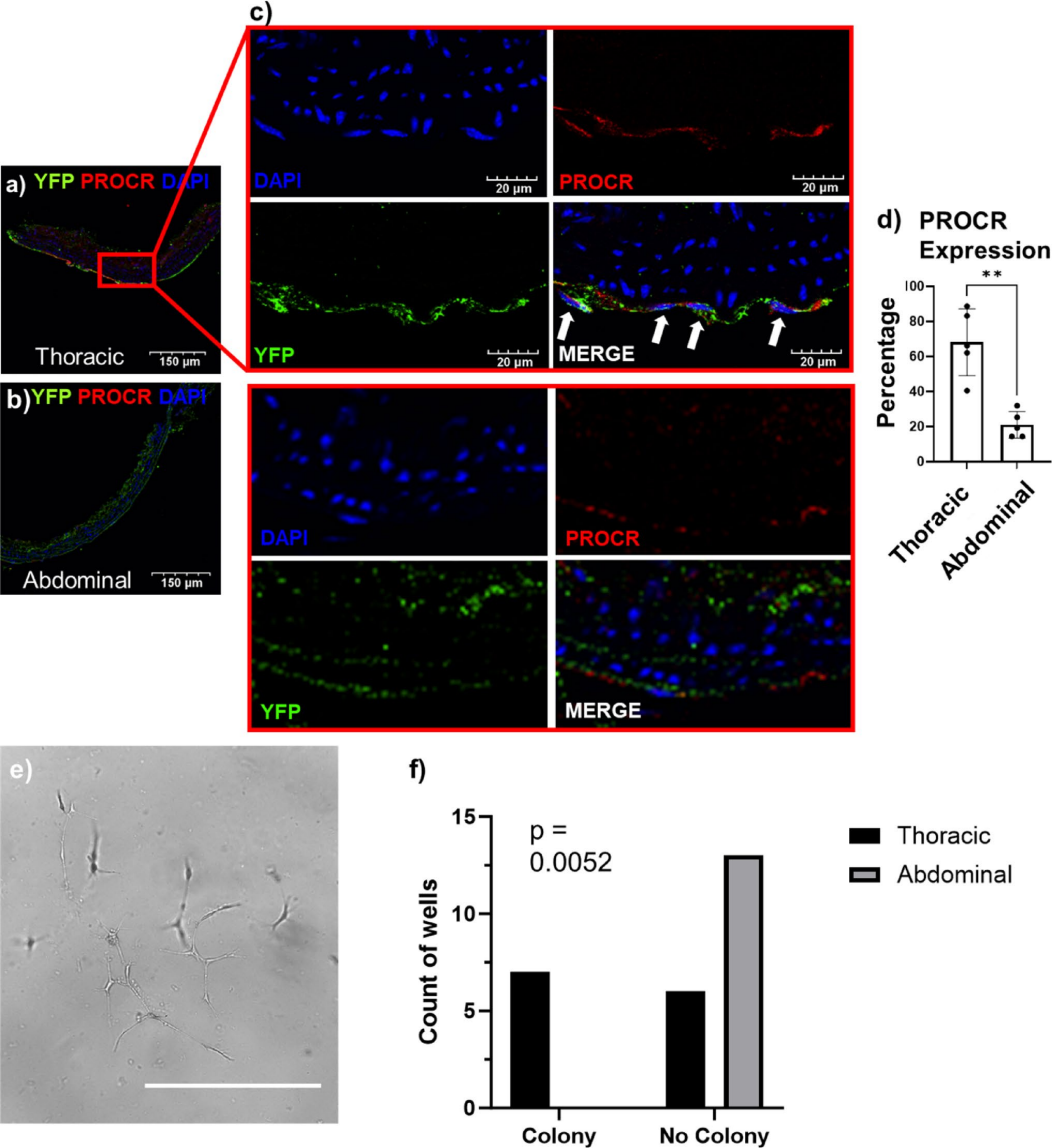
Refined endothelial progenitor cells (rEPCs) form a niche in the thoracic aorta displaying increased clonogenic capacity. **a**–**c** Aortae collected from Cdh5-Cre^ERT2^/ROSA-EYFP mice, opened and rolled lengthwise. **a–b** Representative images of sections of (**a**) thoracic and (**b**) abdominal aorta. **c** Section of thoracic aorta from **A** zoomed to 60x magnification and abdominal aorta from b; white arrows indicate regions of overlap between DAPI, PROCR, and YFP. **d** Quantification showing percent of PROCR^+^ length in abdominal and thoracic aorta (** *p* = 0.005; *n* = 5). **e** Representative brightfield image of colony grown from thoracic aorta of Zs-Green/ROSA-EYFP mice in Matrigel following 12 days; scale bar = 500 μm. **f** Quantification of number of wells that grew colonies from thoracic and abdominal aorta (**, *p* = 0.0052, *n* = 13). Data are presented as mean ± SD. Statistical analysis was performed using paired t-test

In order to confirm that aortic endothelial cells express PROCR and to find their anatomical distribution in situ, aortae from *Cdh5- CreERT2/ROSA-EYFP* mice, treated with tamoxifen to label endothelial cells with YFP, were harvested for ex vivo analysis. Immunofluorescence staining showed greater co-expression of PROCR and YFP in the thoracic aorta (68.18%; Fig. 3a, c and d) as compared to the abdominal aorta (21.10%; Fig. 3b and d; **, *p* = 0.005, *n* = 5).

Next, we investigated if spatial difference of PROCR expression in the aorta [36] also correlated with a spatial difference in functionality in terms of clonogenic capacity. Lin^neg^CD31^lo^CD34^+^YFP^+^ EVPs from both the thoracic and abdominal aortae of *Cdh5- CreERT2/ROSA-ZsGreen* mice were FACS-sorted and plated in Matrigel^®^ to compare the colony forming capacity between the two populations without biasing based on PROCR expression. Interestingly, 7/13 wells containing YFP^+^ EVPs from the thoracic aorta formed classical endothelial colonies while 0/13 wells from the abdominal aorta formed colonies (Fig. 3e-f; **, *p* = 0.0052, *n* = 3) confirming that the thoracic aorta is enriched for rEPCs.

### rEPCs form differentiated endothelial cells in vivo in homeostasis and injury

**Fig. 4:**
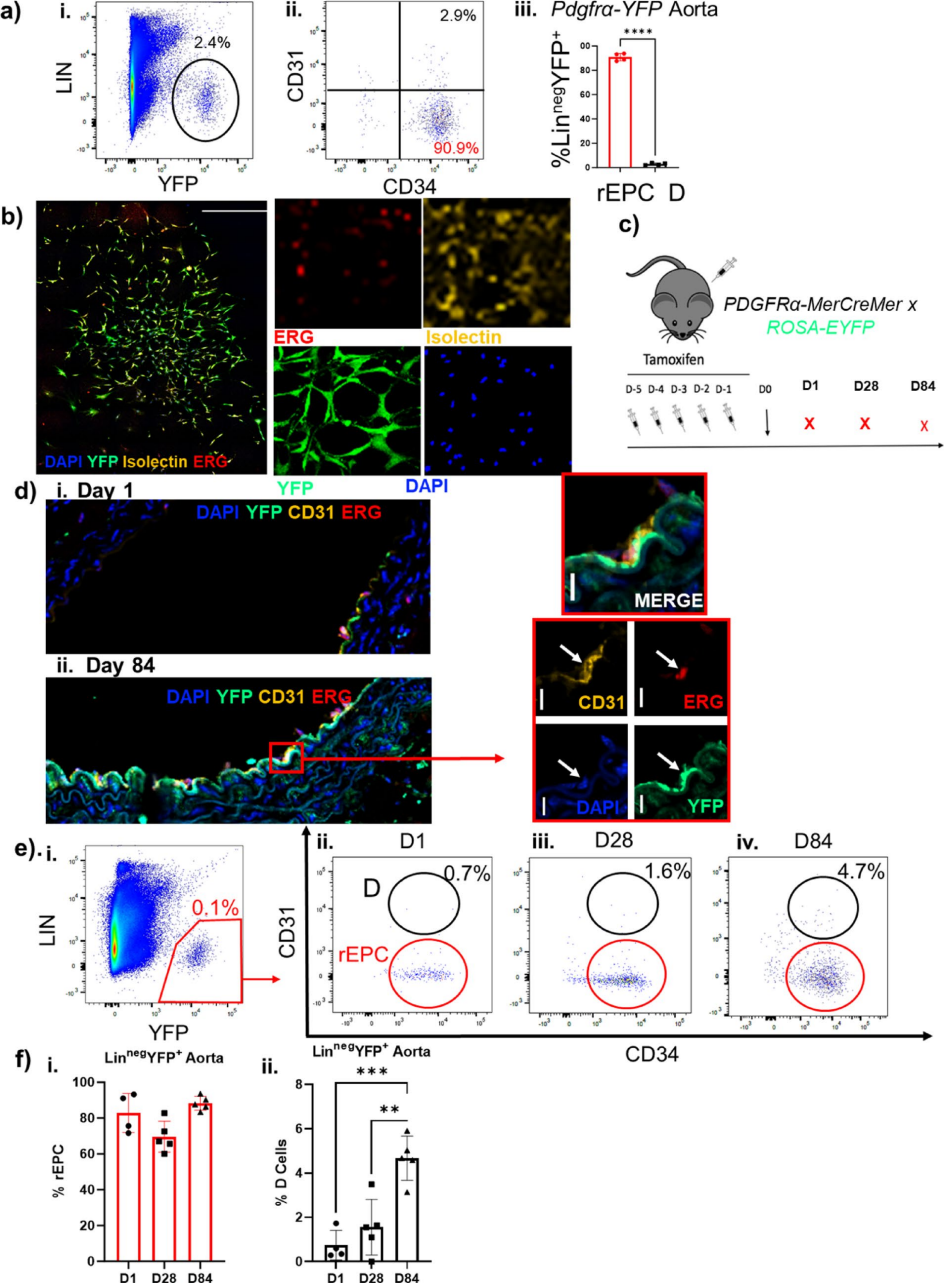
Refined endothelial progenitor cells (rEPCs) from Pdgfra-MerCreMer/Rosa-YFP differentiate into mature endothelial differentiated (D) cells in homeostatic aorta. **a** (**i-ii**) Representative flow cytometry dot plots of PDGFRα-MerCreMer/Rosa-YFP aorta cells showing gating strategy to identify CD34^+^CD31^neg^ endovascular progenitor (EVP) and CD34^+^CD31^+^ D cells from Lineage (Lin)^neg^YFP^+^ population (**iii**) Percent of EVP and D cells in the Lin^neg^YFP^+^ fraction of the adult homeostatic aorta (****, *p* < 0.0001, *n* = 4). **b** Representative image showing immunofluorescence staining of YFP^+^ rEPCs cultured for 12 days from PDGFRα-MerCreMer/Rosa-YFP mice (scale bar = 500 μm). **c** Experimental schematic for aortic lineage tracing where mice are injected with tamoxifen for 5 days prior to commencement of experiment (day (D)0) and collection of tissues at specified timepoints (denoted with a red X). **d** Representative immunofluorescence staining of aorta at D1 (i) and D84 (ii) (large image zoomed in image scale bars = 250 μm, higher magnification images = 10 μm) post cessation of tamoxifen. **e** Representative flow cytometry dot plots showing Lin^neg^YFP^+^ compartment of homeostatic aorta of 4 week old mice injected with tamoxifen changes in proportions of EVPs (**e** (**ii-iv**) shown in red oval) and D cells (**e** (**ii-iv**) shown in black oval) between D1 and D84. **f** Quantification of EVP and D cells in the Lin^neg^YFP^+^ fraction of the aorta between D1 and D84 (** *p* < 0.01, *** *p* < 0.001; *n* = 4). Data are presented as mean ± SD. Statistical analysis was performed using unpaired t-test (a (iii) and ordinary one-way ANOVA (**f**)

PROCR and mesenchymal marker PDGFRA showed a high degree of overlap in rEPCs in flow cytometry and scRNA-sequencing results. Therefore, *Pdgfrα-MerCreMer/Rosa-EYFP* mice were used to trace the fate of rEPCs. Animals were administered with tamoxifen to label PDGFRA-expressing cells permanently with YFP and trace this population in tissues of interest over time. Although the YFP^+^ cells could contain fibroblasts and other populations of mesenchymal origin, flow cytometry analysis of a whole adult homeostatic aorta after a short pulse of tamoxifen, confirmed that the Lin^neg^PDGFRA(YFP)^+^ subpopulation of aorta largely consisted of EVPs rather than differentiated endothelial D cells (Fig. 4a and 91% compared to 3%, *n* = 4, *p* < 0.0001).

To confirm that the population being traced in this model was indeed the same endothelial population as previous studies, YFP^+^PROCR^+^ EVP colonies were cultured from both *Cdh5-Cre*^*ERT2*^*/ROSA-EYFP* and *Pdgfrα-MerCreMer/Rosa-EYFP* aortae. The colonies formed from both models showed no difference morphologically or phenotypically and expressed endothelial markers Isolectin and ERG in vitro (Fig. 4b).

Few studies in the past have been able to identify a single Cre reporter system distinguishing progenitors from differentiated cells given the large overlap in markers. The large differential expression of *Pdgfra* in the aorta between rEPC and differentiated endothelial cells (D cells) provided a unique opportunity to demonstrate the lineage relationship between endothelial populations. Briefly, 4 week-old *Pdgfrα-MerCreMer/Rosa-EYFP mice* were administered with *tamoxifen* to label PDGFRA-expressing cells with YFP (Fig. 4c). Homeostatic aortas were then assessed from juvenile age to adulthood to trace the fate of YFP^+^ cells. Immunofluorescence staining of aorta at day 1 post-tamoxifen (D1) showed YFP^+^ cells in the intima co-expression of ERG and to some extent CD31 (Fig. 4d (i)). This further showed that at least a fraction of PDGFRA-expressing cells labelled by YFP are endothelial as in intimal position and not simply in the mesenchymal layers of the aorta. To examine whether these endothelial cells were rEPCs or of any other subpopulation, we performed flow-cytometry on D1 aorta revealing that all Lin^neg^YFP^+^ cells resided in the rEPC with nearly no fully differentiated D cells (Fig. 4ei and ii, Supplementary Fig. 2c). The fate of D1 labelled *pdgfra*-expressing cells was further examined at D28 and D84. Co-expression of endogenous YFP with CD31 and ERG in the intima at D84 confirmed endothelial fate of these cells and could be identified in some areas of the aorta as patches interrupted by unstained cells, (Fig. 4d (ii)). This result was validated quantitatively using flow cytometry and the percentage of rEPCs between D1 and D84 ranged from an average of 70–88% of the Lin^neg^YFP^+^ compartment, while the percentage of D cells increased significantly from 0.74% at D1 to 4.67% at D84 (Fig. 4e-f; *n* = 5, ** *p* < 0.01, *** *p* < 0.001) suggesting that some rEPCs labelled at D1 gave rise to D cells by D84.

**Fig. 5:**
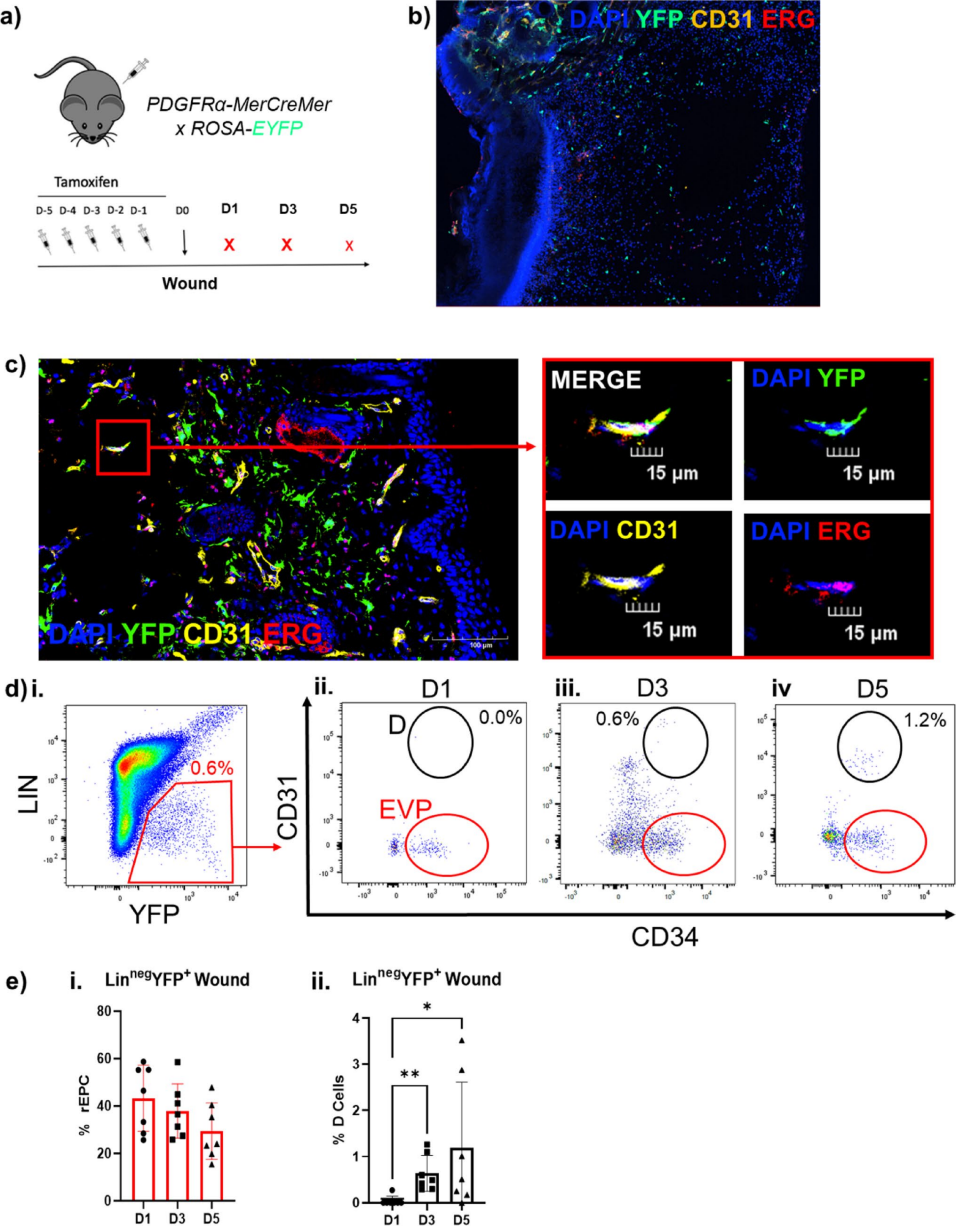
Refined endothelial progenitor cells (rEPCs) from Pdgfrα-MerCreMer/Rosa-YFP differentiate into mature endothelial differentiated (D) cells in an injury model of full-skin excisional wounds. **a** Experimental design for full-skin excisional wound lineage tracing where mice are injected with tamoxifen for 5 days prior to full-skin excisional wounding at day (D)0 and wound site tissue collected at specified timepoints (denoted with a red X). **b-c** Representative immunofluorescence stained images showing skin wound section from D1 (**b**) and D5 with endothelial markers (**c**). **d** Representative flow cytometry dot plots showing lineage (Lin)^neg^YFP^+^ compartment of full-skin excisional wounds from adult mice changes in proportions of endovascular progenitors (EVPs) (**d** (**ii-iv**) shown in red oval) and D cells (**e** (**ii-iv**) shown in black) between D1 and D5. **f** Percent of EVP and D cells in the Lin^neg^YFP^+^ fraction of the wounds between D1 and D5 (* *p* < 0.05, ** *p* < 0.01; *n* = 7). Data are presented as mean ± SD. Statistical analysis was performed using one-way ANOVA (**e**(i)) and Kruskal-Wallis test (**e** (ii))

To analyze this lineage relationship in the context of injury, *Pdgfra-MerCreMer/Rosa-EYFP* mice were treated with tamoxifen before performing full skin excisional wounds at D0 (Fig. 5a). The immunofluorescent staining of the wounds at D1 showed YFP expression on isolated cells in the center of the wounds with no expression of mature endothelial markers as expected (Fig. 5b). This was further confirmed by flow cytometry showing that YFP labelled cells were mostly rEPC (CD31^low/neg^CD34^+^) or mesenchymal (CD31^neg^CD34^neg^) and no D cells. Co-labelling of YFP with CD31 and ERG at D5 demonstrated that YFP^+^ cells have differentiated into mature endothelial cells (Fig. 5c).

Flow cytometry analysis at each time point confirmed that the percentage of rEPCs between D1 and D5 ranged from an average of 29.43–43.29% of the Lin^neg^YFP^+^ compartment, while the percentage of D cells increased significantly from an average of 0.0% at D1 to 1.2% at D5 (Fig. 5d-e, Supplementary Fig, 2d, *n* = 7, * *p* < 0.05; ** *p* < 0.01). Overall, making use of PDGFRA expression as a reporter of rEPCs allowed tracing the fate of this population to demonstrate its contribution to differentiated endothelial cells both in homeostatic aorta and skin wounds.

### PROCR is expressed in human aorta scRNA-seq data and leads to increased clonogenic capacity in a human term placental model of ECFCs

**Fig. 6:**
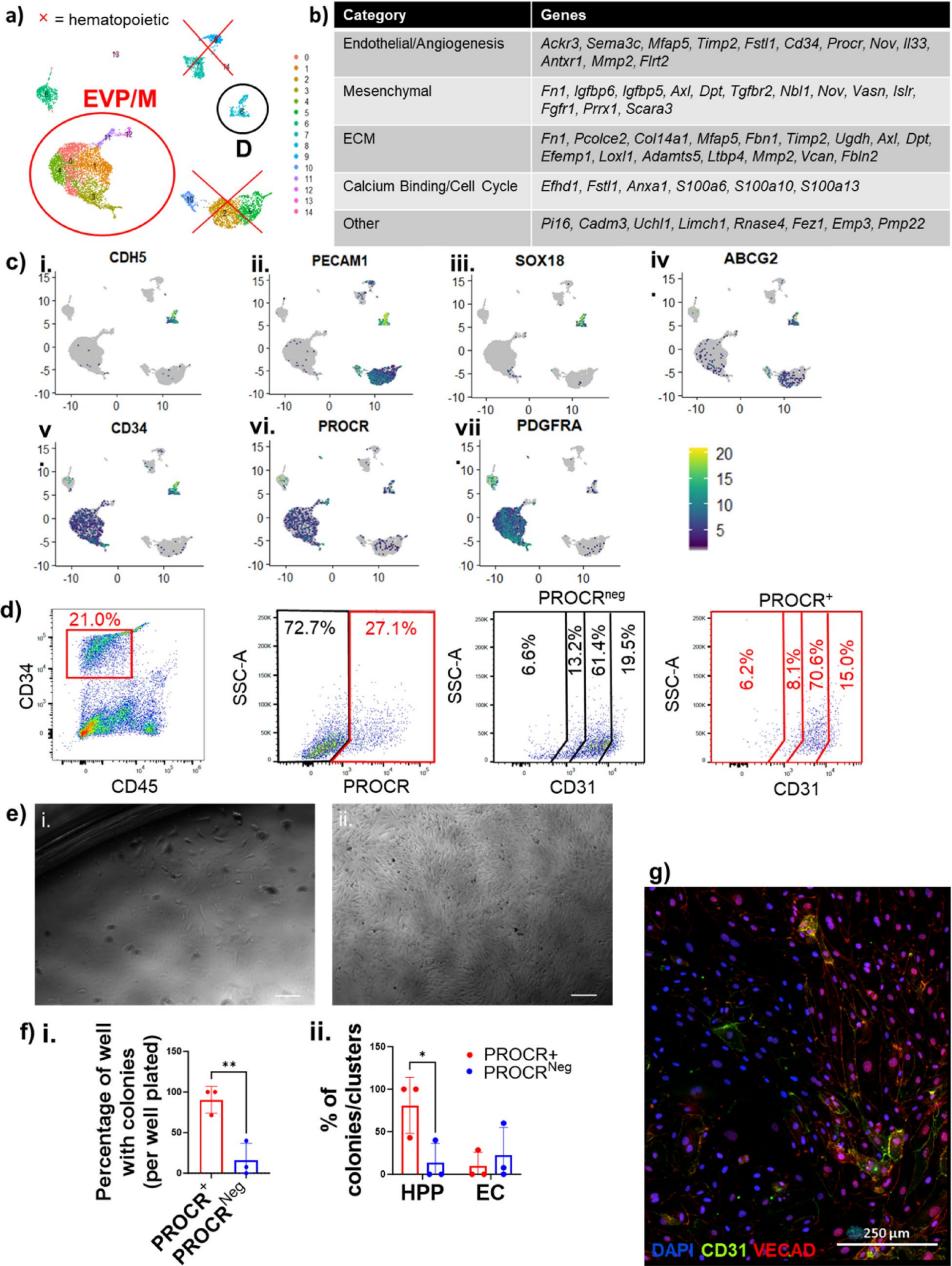
PROCR is expressed in human aorta scRNA-seq data and leads to increased clonogenic capacity in a human term placental model of ECFCs. **a** Single-cell RNA-sequencing data from human control aorta, clustered and filtered to remove hematopoietic clusters and label primary populations (*n* = 3). **b** Categorization of overlapping genes from top 100 DE genes of clusters 3 and 12 from human normal aorta dataset and murine aorta sc-RNA seq endovascular progenitor (EVP) cluster 4. **c** Markers of interest shown across clusters. **d** Representative flow cytometry plots showing the gating strategy of PROCR^+/neg^ endothelial colony forming cells (ECFCs) from human term placenta. **e** Brightfield images of colonies growing from cultured PROCR^neg^ (**i**) or PROCR^+^ (**ii**) ECFCs (scale bar = 200 μm). **f** Quantification of percentage of (**i**) well with colonies and (**ii**) colony types formed from cultured PROCR^neg^ or PROCR^+^ ECFCs. **g** Immunofluorescent staining of PROCR^+^ colony at passage (P)6. Data are presented as mean ± SD. Statistical analysis was performed using unpaired t-test (**f** (i)) and 2 way ANOVA (**f**(ii))

To identify rEPC equivalent population in human tissues, publicly available human normal aorta single-cell RNA-sequencing data was reanalyzed [27]. Data from 3 normal aortae samples was re-clustered after filtering doublets and before removing hematopoietic clusters based on known marker expression and SingleR labeling analysis (Fig. 6a, Supplementary Fig. 3a-b). The remaining clusters were identified based on SingleR labelling as primarily mesenchymal (M). The human counterparts to the rEPC cluster specifically were identified as clusters 3 and 12 as they had high degree of overlap between top differentially expressed (DE) genes in these clusters and rEPC cluster 4 in the murine sc-RNA seq dataset (Fig. 6b, Supplementary Fig. 3b). These overlapping genes were found to be implicated in endothelial, mesenchymal, extracellular matrix, and cell cycle pathways, indicative of genes maintaining a population between endothelial and mesenchymal states (pathways evaluated using EnrichR) [37]. Other genes listed play essential roles in endothelial identity or regulation of mesenchymal transition and fibrosis. Markers of interest outlined above were then analyzed in remaining endothelial, M cell, and EVP-like clusters using FeaturePlots, Dot Plots of top DE genes, and pathway analysis (Fig. 6c, Supplementary Fig. 3c-e).

Major endothelial genes *PECAM* and *CDH5* (Fig. 6c (i and ii)) were most highly expressed in endothelial (D cell) cluster 8 along with *SOX18* and *ABCG2* and (Fig. 6c (iii and iv)). *CD34*, *PROCR*, and *PDGFRA* were expressed in multiple clusters but were highest in EVP clusters 3 and 12 as well as D cell cluster 8 (Fig. 6c (v-vii)).

Upon confirmation that the expression of markers of interest in human control aorta using scRNA-seq resembled the expression seen in mouse models described previously, functional assays were conducted to investigate whether progenitor capacity was increased in human cells expressing these markers as seen in murine studies. Placental CD34^+^CD45^*neg*^ cells were FACS-sorted as described previously [32], with the additional gating of PROCR^+ vs. neg^ for each population of varying CD31 expression (negative, low, intermediate, and high) (Fig. 6d, Supplementary Fig. 3f) and cultured on collagen coated plates. In particular, colony formation of the CD31^lo^ and CD31^int^ populations were examined as both have been shown in the past to contain progenitors that give rise to endothelial colony forming cells (ECFCs) [9]. Given both the CD31 and CD144(*Cdh5*) exhibit highly overlapping expression patterns across human placental endothelial subsets, including endothelial progenitor [9], CD31 alone was used to sort different endothelial populations.

Across all donors, CD31^int^PROCR^+^ cells had 90% more colony forming capacity as compared to CD31^int^PROCR^neg^ cells (15%) (Fig. 6f (i), *n* = 3 placentas). Interestingly, 80% of CD31^int^PROCR^+^ colonies continually expanded and reached the size of high proliferative potential (HPP; >1000 cells) colonies (Fig. 6F (ii)). These HPP colonies were then passaged and showed the ability to continually expand through P6 (Fig. 6g). Moreover, IF staining of these colonies confirmed the endothelial nature of this population with positive expression of CD31 and VE-cadherin (Fig. 6g). Contrarily, CD31^int^PROCR^neg^ cells formed colonies which only grew to < 50 cells before dying, therefore classifying them as endothelial clusters (EC; Fig. 6e (ii), *n* = 3 placentas).

## Conclusion

Here we investigated the expressional and functional overlap of several previously described EPC populations, showing that a degree of uniformity can be uncovered within the heterogenous population of cells that lead to endothelial differentiation and proliferation. Previously identified EVPs show expressional overlap with characterized putative EPC markers PROCR and PDGFRA, both in flow cytometry and in single-cell RNA-sequencing, culminating in a new refined EPC definition (rEPC). Furthermore, gating live murine aortic cells based on positive expression of PROCR and PDGFRA alone was enough to distinguish the rEPC population with ~ 95% accuracy. Further analysis via IF staining revealed that PROCR^+^ endothelial cells mostly reside in the intima of the thoracic aorta and showed increased colony formation capacity compared to abdominal aortic EVPs, supporting the presence of a region-specific endothelial progenitor niche. This may reflect fundamental differences in embryonic origin, local haemodynamic and biomechanical forces, and vascular microenvironmental cues that contribute to a niche-specific signalling cues that preferentially support maintenance of a progenitor-enriched endothelial state within the thoracic intima.

The co-expression of PROCR with other markers of the EVP population is in line with the functional similarities previously shown between EVPs/ECFCs and the PROCR^+^ vascular endothelial stem cell population uncovered by Yu et al., namely the apparent bipotential ability to form cells of both endothelial and mesenchymal potential [9, 16]. A recent study also suggests that PROCR^+^ endothelial progenitors regulate vascular integrity and microenvironment in BM and spleen [38, 39]. Similarly, EVPs were consistently found to be the dominant portion of the YFP^+^ endothelial compartment in the aorta of *Pdgfrα-MerCreMer/ROSA-EYFP* mice compared to D cells. On the contrary, CD157, ABCG2, and SOX18 were all found to be more highly expressed in differentiated endothelial D cells than in EVPs in the murine aorta via flow cytometry, and clustered to a higher degree with D cells than EVPs in single-cell RNA-sequencing. As both CD157 and ABCG2 have been investigated in the context of an E-SP of VESC-like cells, it is possible that this population is distinct from the rEPC population described here [22, 23]. These variations can be due to specific vascular beds and potential differences between the aorta and the liver or other tissues. Additionally, SOX18 may not have been highly expressed in the progenitor population in this context as we have studied the murine aorta in homeostasis, whereas SOX18 has been previously shown to be expressed in the vasculature only under a pathological or developmental stimulus. Therefore, the association of SOX18 with rEPCs might be context dependent and limited to situations of wound healing or tumour growth as reported previously [35].

Beyond expressional confirmation, rEPCs were tested functionally in a series of assays. rEPCs showed increased functional progenitor capacity compared to PROCR^neg^ EVPs or PROCR^+/neg^ D cell populations. rEPCs consistently formed more colonies in vitro than all other populations, as well as being the only population to form strictly endothelial colonies both phenotypically and morphologically.

PROCR^+^ D cells formed 15-fold fewer colonies, with none demonstrating an endothelial phenotype, recapitulating that the overlap of both PROCR and EVP marker expression (Lin^neg^CD34^+^VE-cadherin^+^CD31^lo^) in the endothelium are required to enrich for functional progenitor capacity. It must be acknowledged this assay does not unequivocally assess single‑cell–derived colonies; however, compared with population‑level colony‑forming assays, the limiting dilution approach offers a more refined and standardized evaluation of clonogenic capacity. Moreover, colony formation assays in murine models are more varied that the well-established ECFC assay in human context. Particularly, long term cultures could not be demonstrated here. However, rEPCs from murine aortae showed increased engraftment potential in a 3D collagen matrix in vivo, forming vascular networks within gels embedded in hosts that stained positive for mature endothelial markers after 7 days of implantation, to a significantly higher degree than PROCR^neg^ EVPs while D cells failed to engraft regardless of their PROCR expression. This assay represents a much more robust evaluation of engraftment and regenerative capacity of rEPCs as few as 100 cells could repopulate significant parts of each collagen plug. These experiments clearly highlight the superior engraftment and regenerative capacity of the rEPC population beyond EVP definition or PROCR staining alone.

Further, fate tracing of rEPCs from D1 timepoints in homeostatic aorta and full-skin excisional wounds using *Pdgfrα-MerCreMer/ROSA-EYFP* mice both showed the ability to differentiate into D cells. This formally demonstrates that mesenchymal marker PDGFRA marks a progenitor population capable of endothelial fate in both homeostasis and injury. Particularly, during homeostasis, few reporters can distinguish progenitors from differentiated cells in the endothelium. The intimal position of staining, the flow cytometric gating and the final endothelial fate of the PDGFRA-expressing YFP-labeled cells in the homeostatic aorta clearly point to their endothelial capacity. Given Pdgfrα is not strictly endothelial‑restricted, we cannot absolutely exclude contribution from other PDGFRα⁺ mesenchymal cells. However, our results indicate that the cells labeled at D1 in the homeostatic aorta represent a predominantly endothelial population rather than a mesenchymal source, with this caveat being more applicable to the wound setting. Finally, translation to human models showed that PROCR and PDGFRA are expressed in an EVP-like population in the human normal aorta at the RNA level. Moreover, when further gated on positive PROCR expression, previously defined human term placental ECFCs show increased clonogenic capacity, forming colonies of higher yield and secondary colonies, and self-renewing to at least P6. However, this study still lacks functional validation of human PDGFRA^+^PROCR^+^ cells and will be considered important for future work.

In the present study, PROCR and PDGFRA showed a high degree of overlap within the EVP population, supporting their use in defining rEPCs. While the current assays demonstrated progenitor enrichment within the PROCR+ fraction, additional studies comparing double-positive and single-positive EVP subsets will be needed to determine whether PDGFRA provides further functional enrichment beyond PROCR alone.

Together, these characteristics show that combining the expressional requirements of EVPs with expression of PROCR and PDGFRA characterizes a more specific progenitor population with increased functional capacity within the endothelial compartment of various tissue beds, both in murine and human models. The addition of stringent functional requirements as well as a wider array of cell surface markers allows for EPCs to be targeted more specifically, hereby enhancing mechanistic understanding and providing a preclinical foundation for future vascular regenerative therapies, as well as tissue engineering and bioengineering applications.

## Supplementary Information

Below is the link to the electronic supplementary material.

Supplementary Material 1

## Data Availability

Public single-cell RNA sequencing data of mouse aorta was downloaded from ArrayExpress (https://www.ebi.ac.uk/arrayexpress) under accession number E-MTAB-7149. Public single-cell RNA sequencing data of human aorta was downloaded from the Gene Expression Omnibus (GEO) under accession number GSE155468. No other new sequencing data were generated in this paper.
