# Supplementary material for: Functional definition of endothelial progenitors by PROCR and PDGFRA co-expression
Source: Angiogenesis. 2026 Aug 1;29(4):54. doi: 10.1007/s10456-026-10078-0 (PMC13428779; doi:10.1007/s10456-026-10078-0)
Supplement: Supplementary file 1 — Supplementary Material 1 [file 10456_2026_10078_MOESM1_ESM.pptx]

## Slide 1
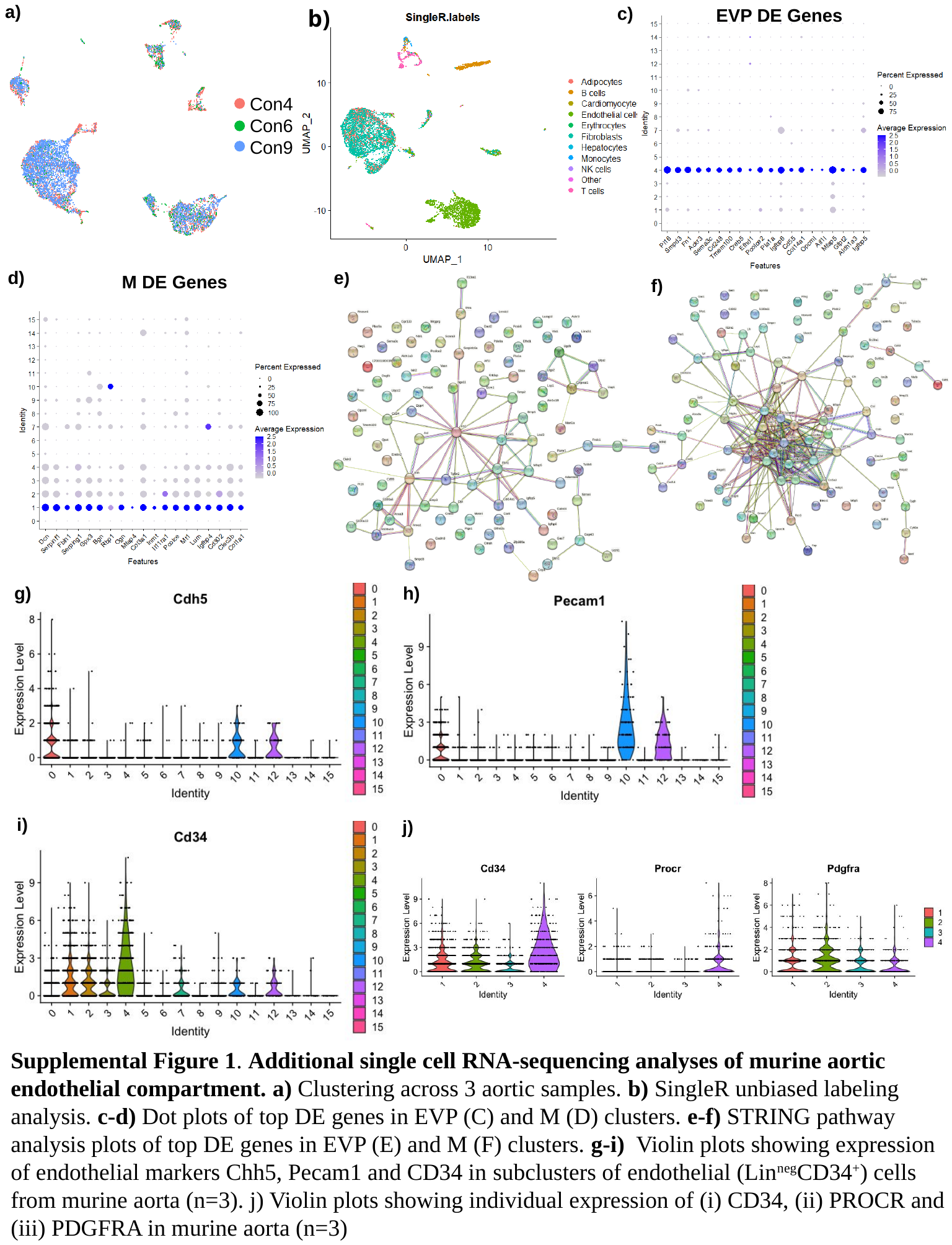

B.
a)
Con4
Con6
Con9
c)
EVP DE Genes
d)
e)
M DE Genes
f)
g)
h)
i)
j)
b)
Supplemental Figure 1. Additional single cell RNA-sequencing analyses of murine aortic endothelial compartment. a) Clustering across 3 aortic samples. b) SingleR unbiased labeling analysis. c-d) Dot plots of top DE genes in EVP (C) and M (D) clusters. e-f) STRING pathway analysis plots of top DE genes in EVP (E) and M (F) clusters. g-i) Violin plots showing expression of endothelial markers Chh5, Pecam1 and CD34 in subclusters of endothelial (LinnegCD34+) cells from murine aorta (n=3). j) Violin plots showing individual expression of (i) CD34, (ii) PROCR and (iii) PDGFRA in murine aorta (n=3)

## Slide 2
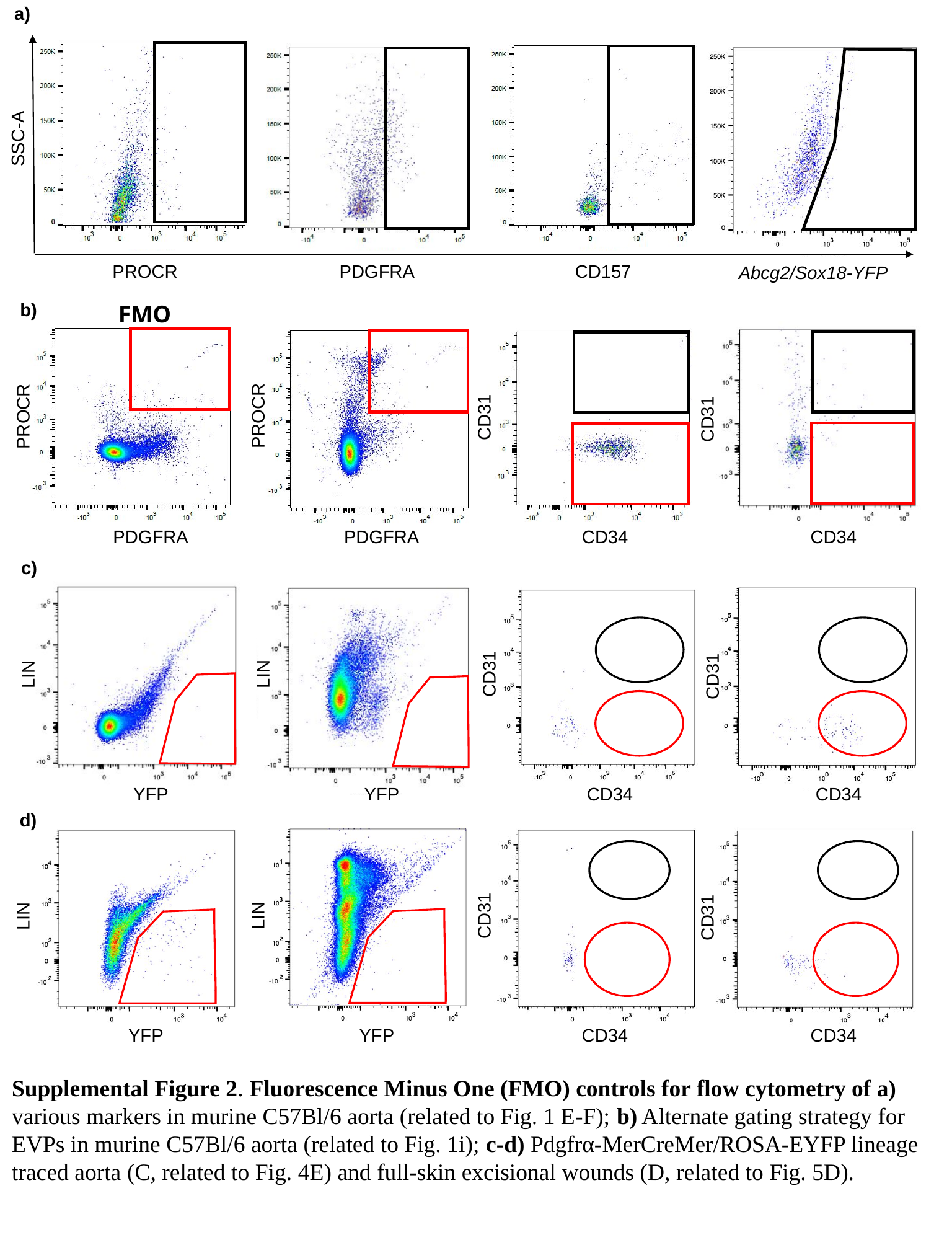

a)
SSC-A
PROCR
PDGFRA
CD157
Abcg2/Sox18-YFP
b)
CD31
CD31
PROCR
PROCR
PDGFRA
PDGFRA
CD34
CD34
c)
CD31
CD31
LIN
LIN
YFP
YFP
CD34
CD34
d)
CD31
CD31
LIN
LIN
YFP
YFP
CD34
CD34
FMO
Supplemental Figure 2. Fluorescence Minus One (FMO) controls for flow cytometry of a) various markers in murine C57Bl/6 aorta (related to Fig. 1 E-F); b) Alternate gating strategy for EVPs in murine C57Bl/6 aorta (related to Fig. 1i); c-d) Pdgfrα-MerCreMer/ROSA-EYFP lineage traced aorta (C, related to Fig. 4E) and full-skin excisional wounds (D, related to Fig. 5D).

## Slide 3
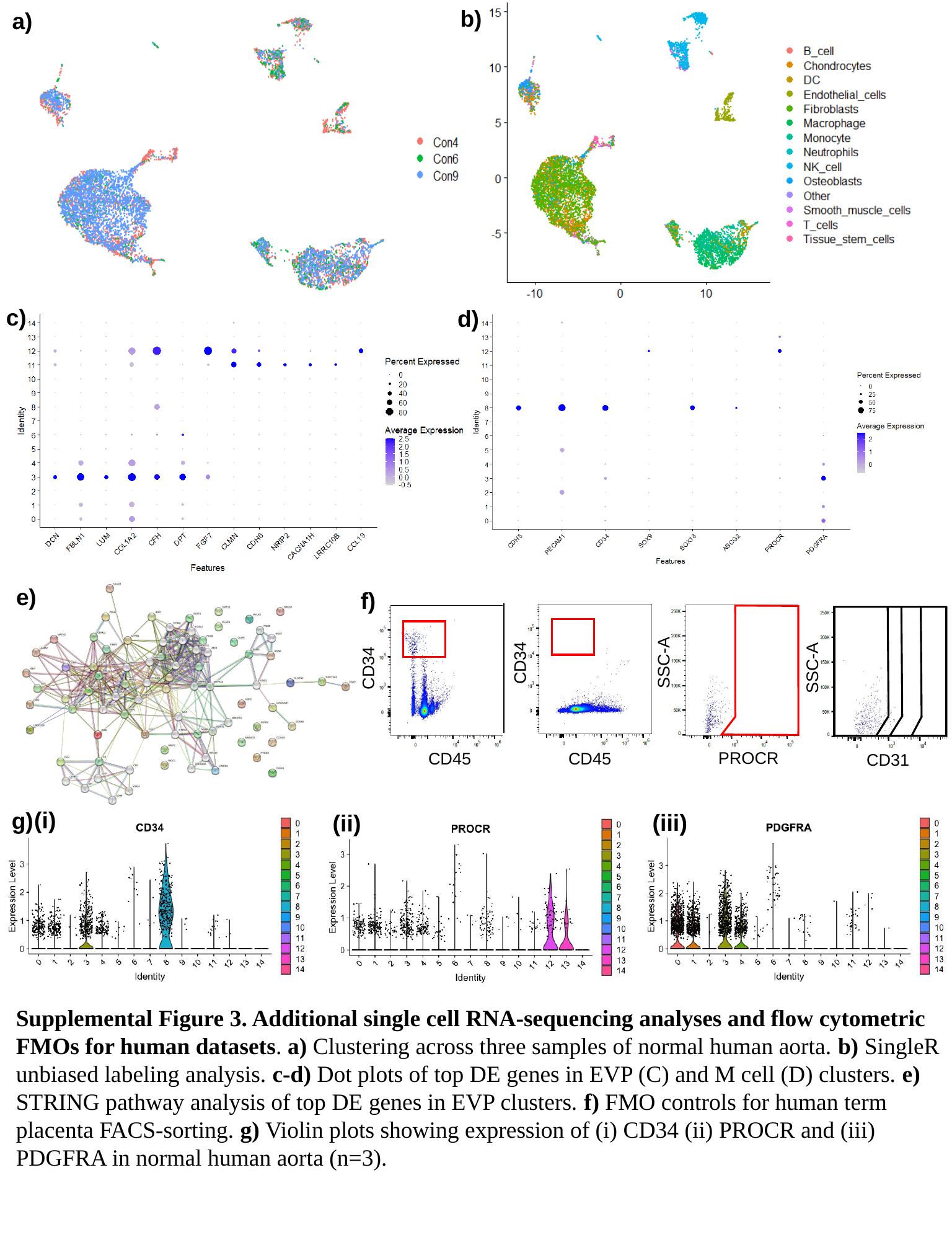

b)
a)
c)
d)
e)
f)
CD34
SSC-A
CD34
SSC-A
PROCR
CD45
CD45
CD31
(i)
g)
(iii)
(ii)
Supplemental Figure 3. Additional single cell RNA-sequencing analyses and flow cytometric FMOs for human datasets. a) Clustering across three samples of normal human aorta. b) SingleR unbiased labeling analysis. c-d) Dot plots of top DE genes in EVP (C) and M cell (D) clusters. e) STRING pathway analysis of top DE genes in EVP clusters. f) FMO controls for human term placenta FACS-sorting. g) Violin plots showing expression of (i) CD34 (ii) PROCR and (iii) PDGFRA in normal human aorta (n=3).
